# Genito-Urinary Surgery

**Published:** 1904-12-31

**Authors:** 


### GENITO-URINARY SURGERY.

*(Concluded from page 227.)*

**Suprapubic Prostatectomy.**—P. J. Freyer[4] has now published an account of 110 cases of enlarged prostate for which he has performed his operation of total extirpation of the gland. He performed the first operation of this kind on December 1, 1900. The relation of the prostate to its fibrous investments have been cleared up by these operations. The author compares the prostate and its coverings to an orange. For the illustration to be more complete, the edible part of an orange may be imagined to consist of two halves, instead of several segments, with the septum between the halves placed vertically. The thin, strong membrane covering the pulp may be taken to represent the true capsule of the prostate, and the rind to represent the outer capsule, or prostatic sheath, formed by the recto-vesical fascia. In the author's operation of prostatectomy the inner, or true capsule, is removed, and the outer capsule, or sheath, is left behind. As the two prostatic lobes enlarge they tend to become less firmly attached to one another at the anterior and posterior commisures, recalling their separate condition in early fœtal life. Also the urethra is loosened from its close attachment to the inner surfaces of the lobes, and for this reason can often be separated from the gland and left behind at the operation. The sheath is incomplete above, and the enlarging upper part of the gland gradually insinuates itself through this opening into the bladder. The bladder-muscle overlying it gets thinner and thinner from pressure, so that eventually the portion of the gland projecting into the bladder is covered by mucous membrane only. The so called middle lobe in all cases is an out-growth from one or both of the lateral lobes. The ages of Freyer's patients have varied from 53 to 84 years; and prostates removed have weighed from ¾ oz. to 14¼ oz. In three instances the disease proved to be malignant, though before operation malignancy was only suspected in one of them. One of the malignant cases died, but the other two made fairly good recoveries. The mortality, apart from the malignant cases, works out at about 9 per cent.

**Perineal Prostatectomy.**—Henry H. Morton, of Brooklyn,[5] has reported 10 cases of this operation. The author accepts the view that senile prostatic enlargement is of inflammatory origin, and in the majority of instances is a remote effect of gonorrhœa. There is a fibroid variety of hypertrophy and an adenomatous form, the former affecting chiefly the connective tissue elements of the gland, the latter the secreting elements. In the latter the inflammation primarily affects the tissues surrounding the excretory ducts, and by compression dams back secretion in the tubules of the gland. The author considers the technique described by Bryson in 1898 to be the best for perineal prostatectomy. Briefly it consists in performing a median urethrotomy, and dividing the floor of the posterior urethra as far as the apex of the prostate. The most prominent portion of the prostatic growth which bulges at the side of the urethra is felt with the finger in the urethra and the mucous membrane over it is punctured with a blunt instrument. Through the rent the forefinger is passed, tearing its way into the centre of the mass. Then the finger is swept round within the capsule of the prostate and the adenomatous tumours are shelled out and extracted with lithotomy forceps. The other side is dealt with in the same way, and finally the middle lobe. A soft rubber catheter is introduced through the wound into the bladder and the wound packed. There were two deaths in 10 cases reported. Two patients were obliged to continue to use a catheter after the operation. In one case a recto-vesical fistula required a subsequent operation for its cure. The remaining five were entirely relieved.

**Gigantic Renal Calculus.**—A. Marmaduke Sheild[6] records a case in which he successfully removed a gigantic renal calculus. The man had had symptoms at intervals for 14 years. For a few days before operation he had had abdominal pain which was getting worse and leading to collapse. Filling the left flank and loin was a large firm swelling. The diagnosis was very dubious and a malignant tumour was suspected. On opening the abdomen a quantity of pus and two small stones escaped from an abscess communicating with the kidney. The kidney was much enlarged and firmly adherent, and contained a stone weighing more than a pound. The stone and the kidney were removed with great difficulty, and pus and urinous fluid poured freely over the intestines in the course of the proceedings. The patient made a good recovery. The stone was five and a half inches long and 10 inches in circumference at its largest part. The man had been able to carry on his work as a cabman until a few days before the operation. The author believes that it is not generally known and appreciated that renal stones, when of great size or very numerous, may form an abdominal tumour of notable size, the nature of which is apt to be misunderstood. An enormous renal stone weighing 36¾ ounces in the St. Bartholomew's Hospital is alluded to. Sheild is inclined to the view that the anterior incision is the best in dealing with this class of case. The results of nephro-lithotomy through the loin are not always encouraging. Septic sinuses are apt to remain in the loin, and the kidney has to be removed later under very disadvantageous circumstances.

[4] Brit. Med. Jour., Oct. 29, 1904, p. 1137, and Lancet, July 23, 1904, p. 197. [5] Med. Record, Oct. 8, 1904, p. 561. [6] Lancet, Oct. 15, 1904, p. 1074.

